# Wound healing potential: evaluation of molecular profiling and amplification of *Lucilia sericata* angiopoietin-1 mRNA mid-part

**DOI:** 10.1186/s13104-020-05141-y

**Published:** 2020-07-01

**Authors:** Hamzeh Alipour, Marziae Shahriari-Namadi, Saeedeh Ebrahimi, Mohammad D. Moemenbellah-Fard

**Affiliations:** 1 Department of Medical Entomology, School of Health, Shiraz University of Medical Sciences, Shiraz, Iran; 2 Research Center for Health Sciences, Institute of Health, Shiraz University of Medical Sciences, Shiraz, Iran

**Keywords:** Wound healing, Therapy, Amplification, *Lucilia sericata*, Angiopoietin, Diptera, Larvae

## Abstract

**Objective:**

High prevalence of chronic ulcers and the burden of disease necessitate the increasingly significant production of new recombinant proteins in the world. The angiopoietin-1 enzyme is a part of the growth factors group which is secreted by *Lucilia sericata* (Diptera: Calliphoridae) larvae when they meet lesions to ensure maggot therapy. It is one of the most potent proteins in wound healing. Given its essential role, the angiopoietin-1 gene of *L. sericata* was characterized, which provided some necessary information on its identity.

**Results:**

The mid-part of the angiopoietin-1 mRNA sequence was thus characterized based on the design of different primers such as exon-exon junction, conserved regions, and specific region primers via conventional polymerase chain reaction (PCR). Its structural features were configured by in silico method. The sequence of mid-part (390 bp) of angiopoietin-1 was determined empirically, and BLAST analysis unraveled its high identity (85%) with the sequence of angiopoietin-1 mRNA of the larval housefly, *Musca domestica*. The homology of this enzyme also exhibited that its nucleic acid sequence was very similar to the domains of angiopoietin-1 in *Lucilia cuprina*. The current data are instructive and critical to evaluate the action of this enzyme in recombinant protein production in future molecular studies on wound healing.

## Introduction

Angiopoietin genes foster wound healing [1]. The angiopoietin-1 enzyme is a portion of the growth factors (GF) group implicated in pre- and neo-natal vessel formation (angiogenesis) and regulating a plethora of cellular and extracellular matrix processes [2]. It involves a multiple of other regulatory roles in the endothelial cells, which are conducive to the cell generation and development of extracellular matrix [3]. A study showed the angiopoietin-1 enzyme had different activities concerning the growth stages involving various cell types while living organisms are faced with wound stress [4]. Two studies have demonstrated that more organ injury and fibrosis arise following elevated, inefficient angiogenesis [5, 6]. The signal of this enzyme is directly exhibited in angiogenesis, as a result of which new blood capillaries form from previous veins and arteries [7]. Angiogenesis enzymes move forward through budding, endothelial cell movement, proliferation, and capillary deformity and stability. They are conducive to assemblage and disassemblage of the interior lining of blood vessels [8]. Angiopoietin-1 cytokines affect the control of microvascular porosity, dilation, and constriction by alarming the smooth muscle cells that have surrounded the capillaries.

Four angiopoietins have been identified as ANGPT1, ANGPT2, ANGPT3, and ANGPT4 [9, 10]. Various extrinsic and intrinsic agents could result in such chronic ailments as diabetic wounds, thermal and radiation burns, injuries, geriatric lesions, and other infection lesions such as leishmaniasis [11], which lead the global prevalence of wounds. These are often chronic and resistant to treatment. Almost 15% of patients with diabetes suffering from foot ulcers, their legs are amputated due to disease complications. That is, a foot is globally amputated due to diabetes every 30 s, while 80% of these cases are preventable [12]. Antibiotic-resistant bacterial infections that cause them are the most problematic subject available at burn centers. Multiple of these microorganisms have long been associated with complications in patients admitted to burn therapeutic centers [13, 14]. Recently, the effect of the angiopoietin-1 has been implicated in the healing of cerebral malaria [15] and eye diseases [16]. Larval (maggot) therapy can be used in the treatment of infectious and diabetic wounds, bedsores, abscessic burns, some types of cancer and bone infection [17]. In 1929, William Beer of Johns Hopkins initiated the first innovation in maggot therapy. In the 1940s, advances in the production and use of the antibiotics reduced the related research about maggot therapy [18]. The performed clinical studies at the University of California in 1989 showed that this method was very effective in improving the wound healing in infections and gangrene [19]. By comparison with the antibiotic-resistant bacterial strains, the maggot therapy is considered as a promising method. *Lucilia sericata* larvae secrete different enzymes which are used in wound healing where one of them is angiopoietin-1. Therefore, maggot therapy is recommended as a selective therapeutic approach due to the diminished risk of damage to the vital organs following infections after surgical operations, speed of treatment, full recovery in patients and the limited use of antibiotics [20].

Further, the use of the *L. sericata* larvae in maggot therapy has been approved by the federal drug administration (FDA) in 2004 [21]. Therefore, the detection and identification of the secreted enzymes by larvae are informative in the design and production of new drugs in the future. One of these enzymes is angiopoietin-1-1, which has a role in angiogenesis and also stimulates growth factors [22].

*Lucilia sericata* distribution is confined to Holarctic and Neotropical zones [23, 24]. Its dispersion in Australia, Colombia, Argentina, Brazil, Chile, and Peru is reported elsewhere [25]. As well as its presence in Iran [26], *L. sericata* is regarded as a synanthropic species close to human residential areas. It is a necrophilic greenbottle insect from the Calliphoridae family [27]. Clinically, two significant effects of larval therapy, including antibacterial compounds secretion and their debridment activities have been ascribed to them [20]. This study aimed to implement the mid-part amplification of angiopoietin-1gene from *L. sericata* larvae as a potential element in wound healing.

## Methods

### *Rearing of Lucilia sericata* larvae

Experiments were conducted on the first instar of *L. sericata* maggots from a colony that had been brought up under constant conditions in the School of Health insectarium, Shiraz University of Medical Sciences (SUMS), Shiraz, Iran. Adult blowflies were encountered to a 12-h L/D cycle at a relative humidity of 40–50% under 18–25 °C. The larvae were nourished with ground chicken liver. Accurate species identification was routinely confirmed using morphological and molecular tools.

### Primer design

**Fig. 1:**
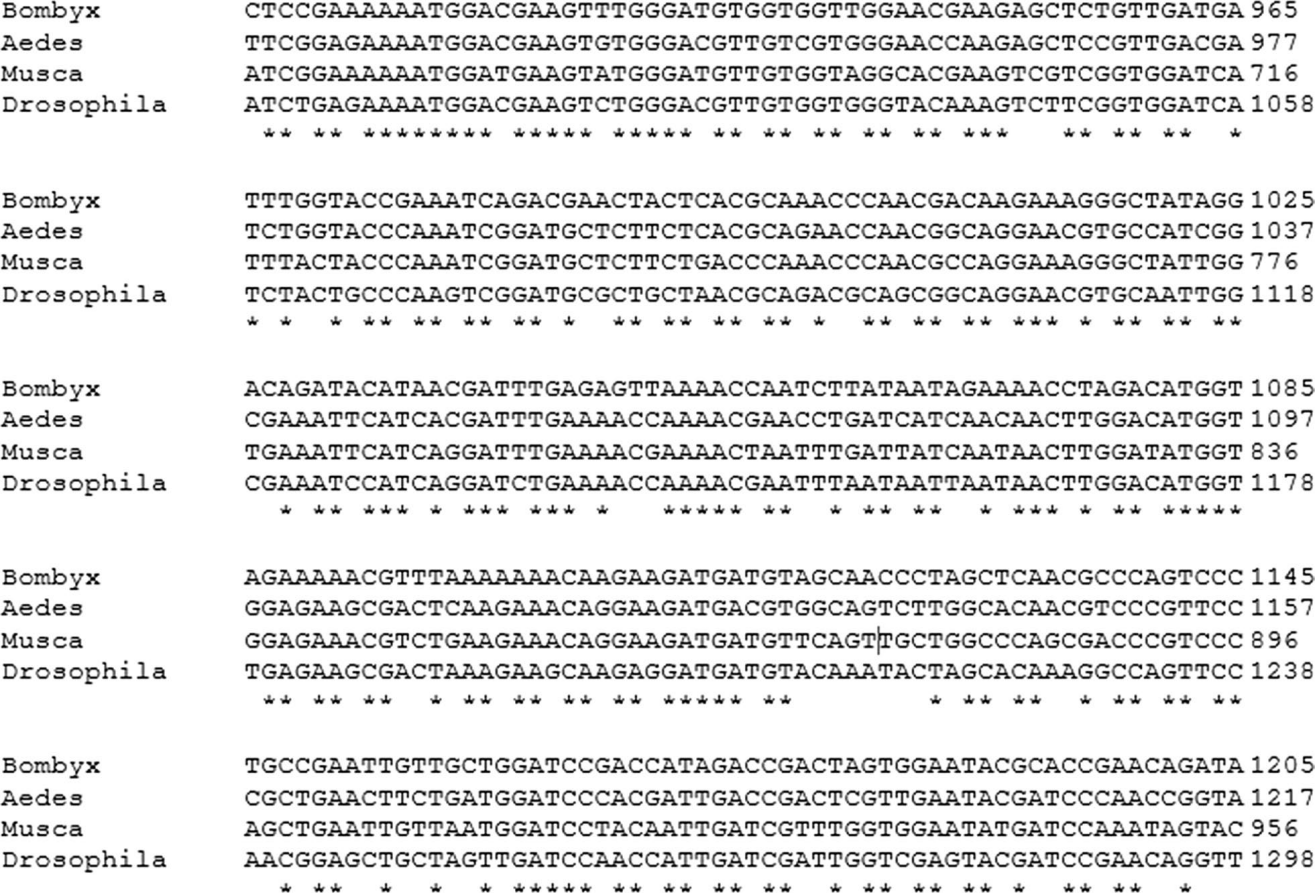
Alignment of angiopoietin-1 mRNA sequences of four insects (*Bombyx, Aedes, Musca,* and *Drosophila*). Sequence comparison was conducted with Clustal Omega based on ClustalW multiple sequence alignment method. Asterisks (*) show conserved sequences

The *L. sericata* genome has not been sequenced yet. Therefore, primers were designed based on our previous studies [20, 28]. The mRNA sequences of Angiopoietin-1 from disparate insects like *Aedes aegypti* (XM_021846146), *Musca domestica* (XM_020038499), *Drosophila arizonae* (XM_018013154) and *Bombyx mori* (XM_004933073), were first obtained from NCBI and aligned using the Clustal Omega computer program (Fig. 1). Following careful consideration, two regions were selected to design gene-specific primers (GSPs). Two exon junction primers AnF1 (5'-AATATATTGGAGTTTATCGG-3') and AnR390 (5'-CGATATACACGAGGCAGTAG-3') were attributed as forward and reverse primers, respectively, to determine the middle part of the target gene. The anticipated amplicon size was 390 bp. Primers were designed by Gene Runner 0.04, Oligo 0.7, and BLAST (online tool) software.

### RNA extraction

Total RNA was extracted from the salivary glands of the third instar cohort of *L. sericata* maggots applying the total RNA purification kit (High Pure RNA Isolation Kit, Roche Company, Germany). The extracted RNAs were treated by DNaseI (Roche Germany), both based on the manufacturer's instructions, and finally stored at −70 °C.

### cDNA synthesis

Extracted RNA was used for the first-strand cDNA synthesis. Then, RT (Reverse Transcription) reaction was done based on the RevertAid First Strand cDNA Syn. kit Fermentas Company by the random hexamers primer.

### Polymerase Chain Reactions (PCR)

All polymerase chain reactions were carried out in a 20 μl total volume for 35 cycles using 2 μl of the synthesized cDNA or 150 ng genomic DNA in each reaction as a template. The reaction mixture included 400 nM of each primer, 1.5 mM MgCl_2_, 1 unit *Taq* DNA polymerase, 0.2 mM dNTPs, 2 μl 10X reaction buffer, and the final volume was adjusted to 20 μl with the double-distilled water (DDW). The amplification program was arranged as follows: 5 min at 94 °C; followed by 35 cycles of denaturation at 94 °C for 30 s, annealing at 58 °C for 30 s, and extension at 72 °C for 80 s; and an extra final extension at 72 °C for 10 min. The amplified amplicons were purified using the DNA gel purification kit (GF-1 Vivantis, Malaysia).

### Sequencing

The normal size amplicons were sequenced after gel purification, and their investigation was implemented by Chromas (Version 2.31, 2005), DNA Star (Version 7.10, 2006), MEGA6 (Build 5110426, 2011) and nucleotide BLAST online website (https://blast.ncbi.nlm.nih.gov/Blast.cgi?PAGE=Protein). Amplicons with the sizes near the predicted range were sequenced using the GSPs forward (AnF) and reverse (AnR390) primers.

### Bioinformatics

Each primer was designed by the Gene Runner (version 0.4) and Oligo 0.7 software. Alignments were implemented by the MEGA software (version 6.0), Clustal Omega online (https://www.ebi.ac.uk/Tools/msa/clustalo/), and their specificity for PCR was vindicated by nucleotide BLAST on NCBI (https://blast.ncbi.nlm.nih.gov/Blast.cgi).

## Results

PCR reactions, which were performed using AnF1and AnR390 on the synthesized cDNA, showed amplification of amplicons near to the expected size of 390 bp, to identify the middle part sequence of *L. sericata* angiopoietin-1 with different primer mixture. The result of the sequencing of the midpart and its BLAST analysis revealed its high identity (85%) with *M. domestica* (XM_020038499.1) angiopoietin mRNA sequences. These steps were undertaken based on our earlier experience and study on *L. sericata* collagenase [20] and *An. stephensi* carboxypeptidase B1 enzymes [29]. The results of this study were stored in GenBank (Accession no: MG009433).

**Fig. 2:**
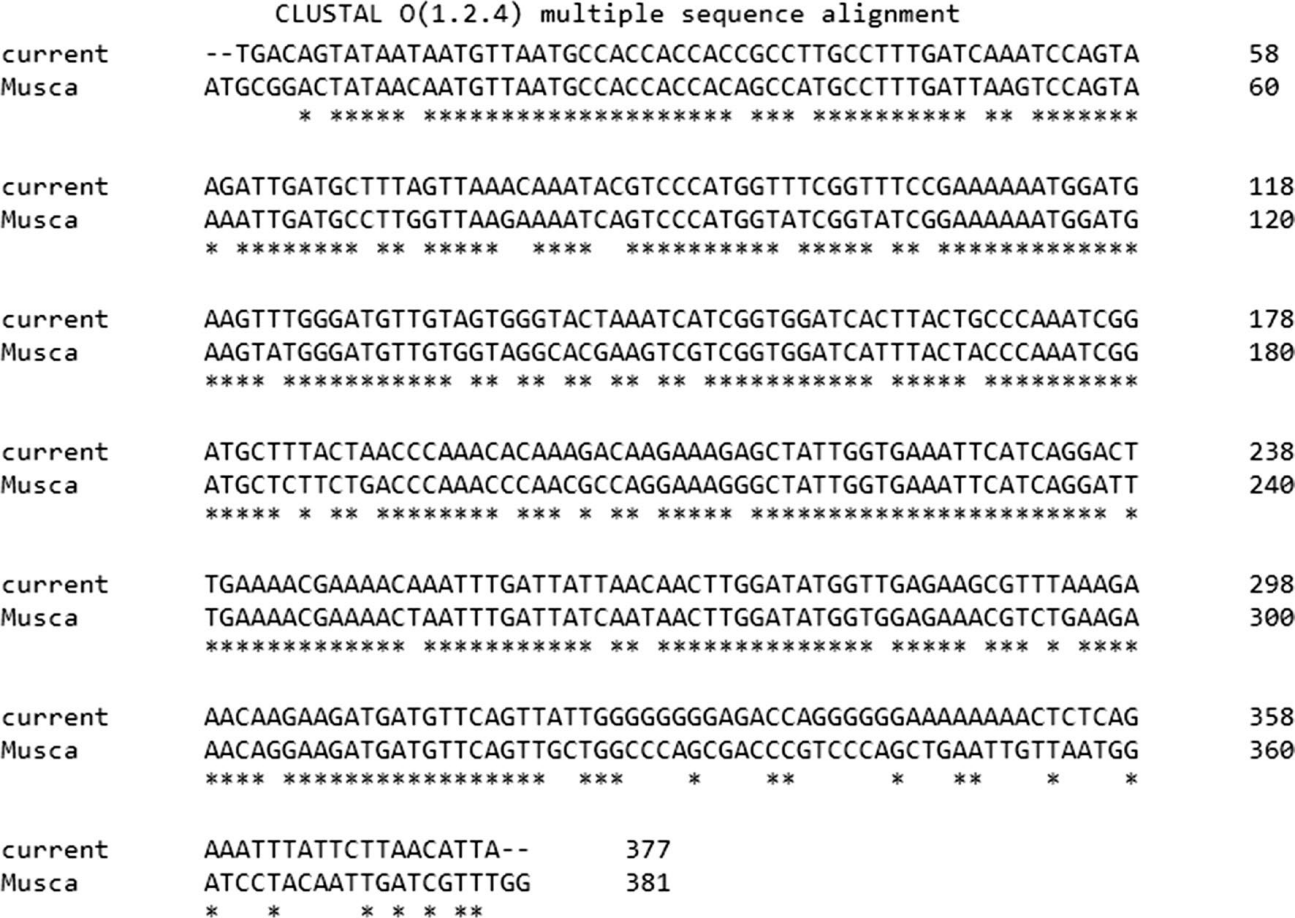
Alignment of the nucleotide sequence of middle region of *Lucilia sericata* angiopoietin-1 (Ang1) mRNA (current) with its counterpart in *Musca domestica* (sequence ID: XM_020038499.1) (*Musca*) using Clustal Omega based on ClustalW multiple sequence alignment method (https://www.ebi.ac.uk/Tools/msa/clustalo/). The Conserved residues have been asterisked

The total alignment of the middle part of angiopoietin-1 mRNA in the GeneBank is shown below (Fig. 2). It has the highest similarity with *M. domestica* (Sequence ID: XM_020038499.1) and *Drosophila arizona* (XM_018013154.1) by maximum identity of 85% and 78%, respectively.

Eventually, the partial sequence of angiopoietin-1 mRNA was characterized in this study that includes the coding sequence of the active site of angiopoietin-1 of *L. sericata*. Therefore, it would enable us to use this sequence to identify the 3' and 5' region of full sequences of angiopoietin-1 mRNA in the future to produce the relevant recombinant protein that could be used in wound healing.

**Fig. 3:**
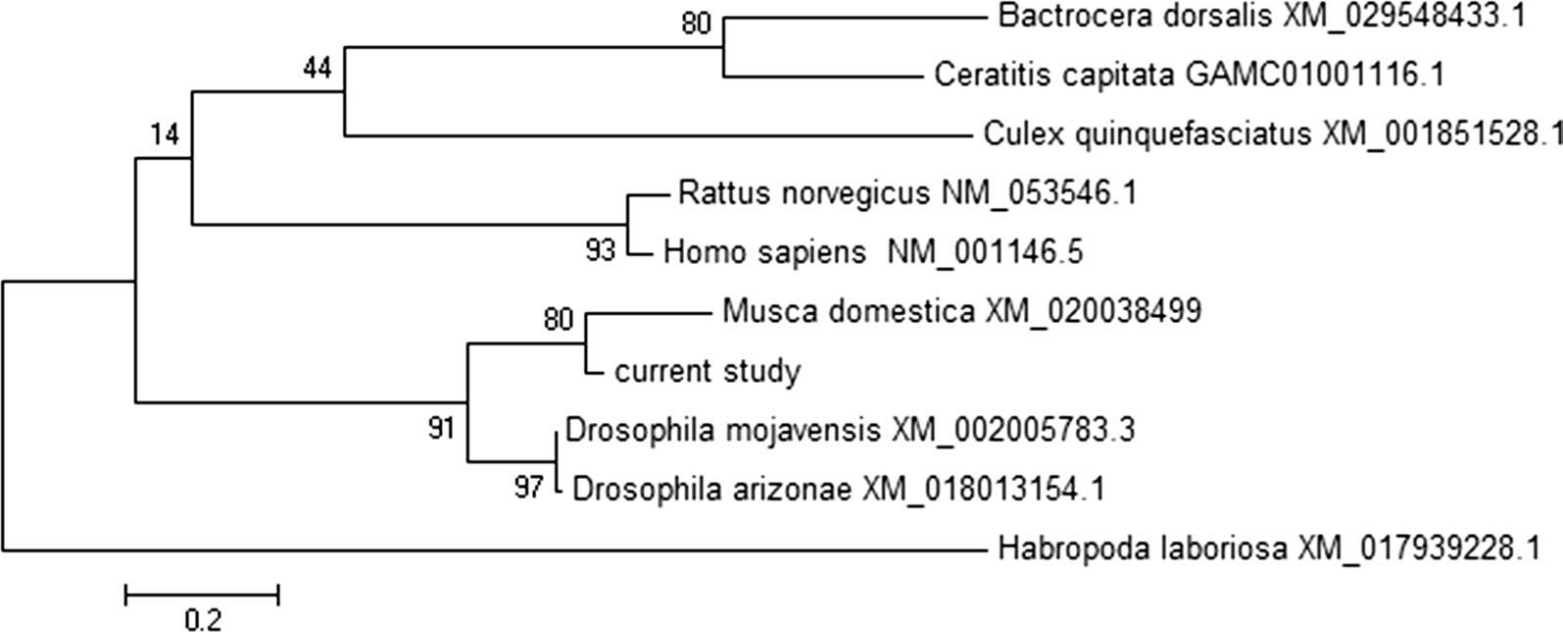
Phylogenetic tree of the characterized Angiopoietin-1 (Ang1) genes of some insects and *Homo sapiens*. Characterized Ang1 genes of other insects and human were aligned and compared by MEGA6.0 software based on Neighbor Joining method with bootstrapping to provide confidence for phylogeny tree. The accession number of each sequence has been presented in a bracket in front of its name. The percentage of replicate trees in which the associated taxa clustered together in the bootstrap values (1000 replicates) has been shown next to the branches. The scale bar corresponds to 0.2 changes per nucleotide

A phylogenetic tree was depicted based on the angiopoietin-1 nucleic acid sequences of four fly species by the Maximum Likelihood method (Fig. 3). The maximum-likelihood bough lengths are calculated for these variant tree topologies and the highest likelihood protected as the best election yet. The phylogeny tree was drawn by using the MEGA 6.0 software. The angiopoietin-1 sequence had the most homogeneity to *M. domestica* (XM_020038499) and *D. arizona* (XM_018013154) nucleic acid sequences.

## Discussion

The current research finding is that maggot secretion includes the differential proteins such as the groups of serine proteinase, metalloproteinase, cysteine proteinase, and growth factors that are useful in wound healing [25]. Angiopoietin is present in the family of vascular growth factors. Tan and Chong demonstrated that the dermal treatment of the recombinant protein of angiopoietin-like 4 (ANGPTL4) promoted wound healing in mice afflicted with diabetes [1]. This conclusion is according to the molecular characterization of angiopoietin-1, which was detected for the first time from salivary glands of stage 3 *L. sericata* larvae. Proteomic studies showed that *L. sericata* larvae secretions have three main ingredients, including proteases, small antimicrobial peptides, and growth factors [30]. Salivary glands secretions are a very favorable source of candidates for development of the new recombinant proteins that are applicable in the treatment of diseases, such as bedsore, burns, osteomyelitis, diabetic foot ulcer, cystic fibrosis, and chronic wounds [31–33]. Maggot debridement therapy (MDT) was currently resurrected due to the appearance of highly specific modifying conditions, particularly the emergence of antimicrobial resistance, which induced reinitiation of the search for optional methods to cure the chronic wounds. In this study, the middle-part sequence of angiopoietin-1 was determined. A patent has been published for the angiopoietin-like 4 (ANGPTL4) and a method of its use in wound healing [1]. It has recently been determined that ANGPTL4 is a matricellular protein involved in the regulation of metabolism and wound healing process and present in the extracellular matrix (ECM) [34]. Goh et al. demonstrated that the deficiency of ANGPTL4 in mice resulted in impeded wound re-epithelialization, diminished expression of ECM proteins, increased skin inflammation, and damaged ulcer-related angiogenesis [34]. The studies showed that 79 genes, whose interim expression portrait throughout wound healing could be grouped into their bioactive theme like cell migration, angiogenesis, proliferation, inflammation, and cell apoptosis [1]. This study succeeded in amplification of the middle part of the angiopoietin gene firstly from *L. sericata* that is the most critical insect in maggot therapy procedure. Angiopoietin, in addition to a few other enzymes like collagenase proteins, is the primary candidate in the production of recombinant protein drugs. The obtained results from the current project provide the prerequisite genomic data of angiopoietin in a famous fly with its therapeutic challenge, which would promote the designing of active new recombinant protein, an essential component for wound healing. Recently, a patent has shown that topical application of angiopoietin-4, particularly the C-terminal fibrinogen-like domain (angiopoietin-4), speeds wound closure of splint-wound model in diabetic ulcer in mice and reduces the deposit of collagen scarring at the remodeling stage of wound healing. The angiopoietin-like-4 can also be employed as an antiulcer factor [35, 36]. To conclude, we successfully identified the mid-part of angiopoietin-1 gene in *L. sericata* Iranian species. This research is the first report and bioinformatics analysis of the mentioned gene in *L. sericata* that could be applied as a basis for full sequence detection of this gene in future studies.

## Limitations

The timing of gene expression for the extraction of RNA, which contains the expressed gene angiopoietin, was one of the research problems. It is possible to go onwork and identify the full gene by the RACE method with the identification of the middle part in this study, which the authors are searching. Another drawback was that only a small region of the gene was characterized, so no functional characterization was plausibly performed.

## Data Availability

The datasets used and/or analyzed during the current study are available from the corresponding author on reasonable request.

## Abbreviations

ANGPT1-4: Angiopoietin types 1-4
ANGPTL: Angiopoietin-like
BLAST: Basic local alignment search tool
cDNA: Circular deoxyribonucleic acid
DDW: Double distilled water
dNTP: Deoxynucleoside triphosphate
ECM: Extracellular matrix
FDA: Federal drug administration of USA
GF: Growth factor
GSP: Gene-specific primer
L/D: Light/dark
MDT: Maggot debridment therapy
mRNA: Messenger ribonucleic acid
PCR: Polymerase chain reactions
RT: Reverse transcription
